# A survey on multi-objective recommender systems

**DOI:** 10.3389/fdata.2023.1157899

**Published:** 2023-03-22

**Authors:** Dietmar Jannach, Himan Abdollahpouri

**Affiliations:** ^1^Department of Artificial Intelligence and Cybersecurity, University of Klagenfurt, Klagenfurt, Austria; ^2^Spotify, Inc., New York, NY, United States

**Keywords:** recommender systems, evaluation, multistakeholder recommendation, beyond-accuracy optimization, short-term and long-term objectives

## Abstract

Recommender systems can be characterized as software solutions that provide users with convenient access to relevant content. Traditionally, recommender systems research predominantly focuses on developing machine learning algorithms that aim to predict which content is relevant for individual users. In real-world applications, however, optimizing the accuracy of such relevance predictions as a single objective in many cases is not sufficient. Instead, multiple and often competing objectives, e.g., long-term vs. short-term goals, have to be considered, leading to a need for more research in multi-objective recommender systems. We can differentiate between several types of such competing goals, including *(i)* competing recommendation quality objectives at the individual and aggregate level, *(ii)* competing objectives of different involved stakeholders, *(iii)* long-term vs. short-term objectives, *(iv)* objectives at the user interface level, and *(v)* engineering related objectives. In this paper, we review these types of multi-objective recommendation settings and outline open challenges in this area.^1^

## 1. Introduction

Generically defined, recommender systems can be characterized as *software solutions that provide users convenient access to relevant content*. The types of conveniences that such systems provide can be manifold. Historically, recommender systems were mainly designed as information filtering tools, like the early GroupLens system (Resnick et al., 1994) from 1994. Later on, various other ways were investigated how such systems can create value, e.g., by helping users *discover* relevant content, by providing easy access to related content (e.g., accessories), or by even taking automatic action like creating and starting a music playlist.

While recommender systems can serve various purposes and create value in different ways (Jannach and Zanker, 2021), the predominant (implicit) objective of recommender systems in literature today can be described as “guiding users to relevant items in situations where there is information overload,” or simply “finding good items” (Herlocker et al., 2000; Manouselis and Costopoulou, 2007; Cacheda et al., 2011; Kamishima et al., 2018). The most common way of operationalizing this information filtering problem is to frame the recommendation task as a supervised machine learning problem. The core of this problem is to learn a function from noisy data, which accurately predicts the *relevance* of a given item for individual users, sometimes also taking contextual factors into account.

Although the actual relevance of recommended items can be assessed in different ways (Gunawardana and Shani, 2015), data-based offline experiments dominate the research landscape. In the early years, rating prediction was considered a central task of a recommender, and the corresponding objective was to minimize the mean absolute error (MAE), see Shardanand and Maes (1995) for work using MAE in 1996. Nowadays, item ranking is mostly considered to be more important than rating prediction, and a variety of corresponding ranking accuracy measures are used today.

While the metrics changed over time, the research community has been working on optimizing relevance predictions in increasingly sophisticated ways for almost 30 years now. The main objective of such research is to minimize the relevance prediction error or to maximize the accuracy of the recommendations. The underlying assumption of these research approaches is that better relevance predictions lead to systems that are more valuable for their users. This seems intuitive for many practical applications because a better algorithm should surface more relevant items in the top-N lists shown to users.

Such an assumption might however not always be true, and it was pointed out many years ago that “being accurate is not enough” (McNee et al., 2006) for a recommender system to be successful. A recommender system might for example present users with obvious recommendations, e.g., recommending new Star Wars sequels to a Star Wars lover. The prediction error for such recommendations might be even close to zero. But so will the value of the recommendations to users, who most probably know these movies already. Observations like this led to a multitude of research efforts on “beyond-accuracy” measures like diversity, novelty, or serendipity, see Bradley and Smyth (2001) for an early work from 2001.

Such beyond-accuracy measures typically compete with accuracy measures (Shi, 2013; Isufi et al., 2021), leading to the problem that multiple objectives have to be balanced when serving recommendations. Which beyond-accuracy dimensions are relevant for a given setting and how much weight should be given to the competing objectives in practice depends on application-specific aspects and in particular on the purpose the recommender is intended to serve (Jannach and Adomavicius, 2016).

Historically, when considering the purpose of a recommender system, the focus of the research was on the value of such a system for *consumers*. Only in recent years, more attention has been paid to the fact that recommender systems in practice factually serve some business or organizational objectives. Considering these platform and item provider-side aspects, therefore, requires that we see recommendation as a problem where the interests and objectives of multiple stakeholders must be considered (Abdollahpouri et al., 2020; Abdollahpouri and Burke, 2022), often also taking different optimization time horizons into account. In Abdollahpouri et al. (2020), the authors emphasize different types of stakeholders in a recommendation environment, namely, consumers, providers, and the recommendation platform. Plus, there can also be side stakeholders such as society. An ideal recommender system operating in a multi-stakeholder environment should aim to balance the objectives of different stakeholders to ensure all stakeholders are satisfied to a certain extent.

Overall, while being able to predict the relevance of individual items for users remains to be a central and relevant problem, considering only one type of objective, i.e., prediction accuracy, and the corresponding metrics may be too simplistic and ultimately limit the impact of academic research efforts in practice. Unfortunately, while we observed an increased research interest in beyond-accuracy metrics during the last 10 years, a large fraction of published works today focuses exclusively on accuracy or a rather limited set of other quality-related metrics. Therefore, one important way to escape the limitations of current research practice is to consider multiple types of optimization goals, stakeholder objectives and their trade-offs in parallel (Jannach and Bauer, 2020). Next, in Section 2, we will discuss various forms of multi-objective recommender systems found in the literature. To the best of our knowledge, the taxonomy we provide in this paper is the first in giving a holistic view of the landscape of multi-objective recommender systems. A recent survey on the topic by Zheng and Wang (2022) focuses largely on the specifics of existing technical approaches to balance multiple optimization objectives and discuss which approach is suitable for which class of problems. We refer readers to this valuable survey on technical aspects. Our present work in contrast aims to provide a more holistic picture of the various forms of multi-objective recommendation problems.

## 2. A taxonomy of multi-objective recommendation settings

In this section, we will first provide a high-level overview of a taxonomy of multi-objective recommendation settings and then discuss the individual components and representative examples in more depth.

### 2.1. Definition and taxonomy overview

**Figure 1:**
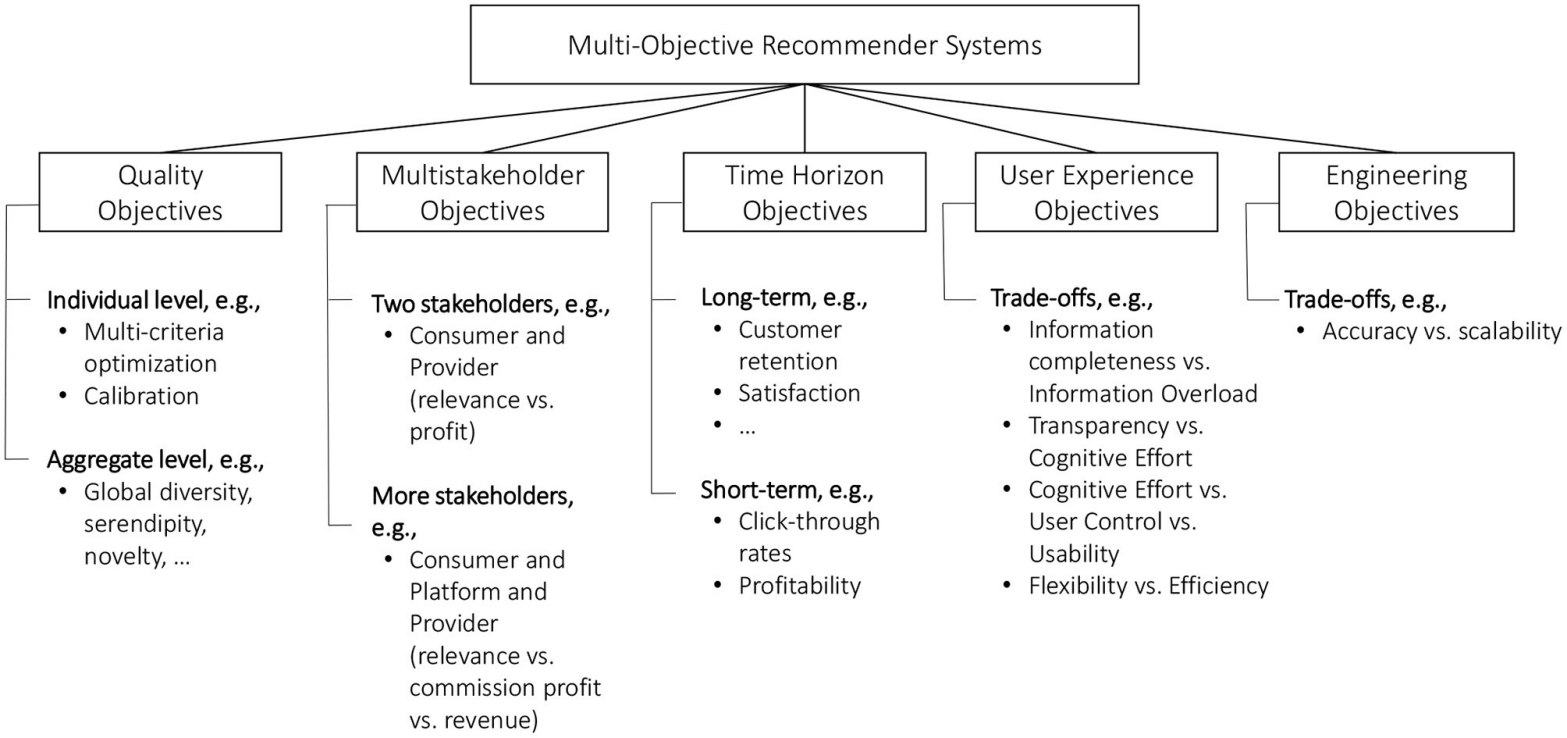
Taxonomy of different types of multi-objective recommendation settings.

On a very general level, we can define that “*a multi-objective recommender system (MORS) as a system designed to jointly optimize or balance more than one optimization goal*.” Figure 1 provides a taxonomy of different types of multi-objective recommendation settings.

We differentiate between five main *types* of objectives:

- **Quality objectives:** Various aspects that can contribute to the quality of recommendations presented to users, including relevance (accuracy), diversity, or novelty. In many cases, these quality objectives are assumed to be competing.
- **Multistakeholder objectives:** Recommender systems are usually designed with the goal of creating value both for consumers, service providers (also called *recommendation platforms*), and maybe other stakeholders such as item suppliers. Challenges for example arise when the best (most relevant) recommendations for the consumer are not the most valuable ones from the perspective of other involved stakeholders.
- **Time horizon objectives:** Recommendations can both impact the short-term and the long-term behavior of users. In the short term, recommendations are designed to help users to find relevant content and/or to influence their choices. Recommendations can however also have longitudinal effects, both positive ones (such as trust building toward the platform) or negative ones (such as filter bubbles) (Pariser, 2011), and again, long-term and short-term objectives may be competing.
- **User experience objectives:** There are various design options and potential trade-offs when developing the user experience of a recommender system. For example, one might try to reduce the cognitive load for users by limiting the amount of information that is presented, e.g., in terms of the number of choices. On the other hand, some users, sometimes referred to as “maximizers” (Schwartz et al., 2002) may instead prefer to see the full spectrum of options before making a decision.
- **Engineering objectives:** Finally, there may be trade-offs regarding engineering (or: system) related aspects. Modern machine learning models can for example be costly to train and challenging to debug. In such situations, it has to assessed if the investments in more complex solutions pay off in practice.^2^

We emphasize that the objectives in the described categories are not mutually exclusive, and in many cases, there are dependencies between the objectives in practice. This may not be immediately apparent from the academic literature, which historically largely focuses on quality objectives. In practical settings, however, the impact of the recommendations on the relevant Key Performance Indicators (KPIs) of the recommendation service provider will almost always be part of the optimization objectives as well.

Moreover, as mentioned, in many cases, the objectives both within a category and across categories can be competing and represent a trade-off. Dealing with such trade-offs is a common target in academic literature, in which most evaluations are done offline, i.e., based on historical data and without users in the loop. In such settings, the goal is then to find a balance between two or more computational metrics, e.g., diversity and accuracy. Limited research unfortunately exists that examines potential trade-offs through real-world experiments. The simulation study in Mehrotra et al. (2018) is an example of a work that is based on real-world A/B test log data, which indicates that increasing the system's fairness may lead to higher user satisfaction and engagement in practice. Also, when considering short-term and long-term objectives, taking measures to increase interactivity and engagement with the system in the short term is sometimes considered beneficial for customer retention in the long run (Gomez-Uribe and Hunt, 2015).

We discuss the different elements of our taxonomy and selected representative works next.

### 2.2. Recommendation quality objectives

Under this category, we subsume problem settings where more than one quality objective of recommendations for users must be considered. We can differentiate between the system considering such objectives at the level of individual users or at an aggregate level, i.e., for the entire user base.

#### 2.2.1. Individual level

At the individual level, consumers can have specific (short-term) preferences, e.g., regarding item features that should be considered in parallel. For instance, a user of a hotel booking platform might be interested in a relatively cheap hotel, which in addition is in close proximity to the city center. In such a situation, the user has multiple criteria in mind for picking the ideal item and the goal of the recommender system is to balance these criteria and recommend items to the user that match the desired criteria as much as possible.

A central problem for a recommender system in such situations is to acquire or derive the user's preferences for the different dimensions. In many cases, and in various early systems like the 1997 “FindeMe” approach to assisted browsing (Burke et al., 1997), preference elicitation is done in an *interactive* or *conversational* approach, see Gao et al. (2021); Jannach et al. (2021) for recent surveys on the topic. The acquisition of the user preferences can be done in different ways, e.g., through pre-defined dialog paths (e.g., Jannach, 2004), through statically or dynamically proposed *critiques* on item features (e.g., Chen and Pu, 2012), or, as done in most recent works, through natural language interactions (e.g., Li et al., 2018). A variety of alternative approaches were proposed as well, e.g., based on the Analytic Hierarchy Process (AHP), e.g., Liu and Shih (2005).^3^ On a general level, such interactive recommendation systems, therefore, support their users in a Multi-Criteria Decision-Making (MCDM) process (Triantaphyllou, 2000; Manouselis and Costopoulou, 2007).

Various technical approaches can be used to derive a set of suitable recommendations once the preferences are acquired. In *constraint-based* systems, for example, explicitly specified rules are commonly used which filter out items that do not match the user preferences. In *case-based systems*, similarity functions play a central role in item retrieval. And in natural-language based systems sentiment analysis can for example be used to derive the user's preferences toward certain items or item features, and these preferences may then be fed into a collaborative filtering algorithm (Smyth, 2007; Felfernig et al., 2015; Li et al., 2018). In particular, in the case of constraint-based systems, the situation may occur that none of the items in the catalog fulfills all specified preferences. For example, assume a user is only interested in hotel rooms cheaper than $100 per night and in less than 5 kilometers from the city center. If no hotel room matches such constraints, the algorithm can relax some of the constraints so a set of recommendations that partially matches the user's criteria can be returned (Felfernig et al., 2015). Furthermore, methods like Multi-Attribute Utility Theory can be applied to rank the remaining candidates (Huang, 2011).

Besides approaches that interactively acquire the user preferences regarding certain item features, another line of research exists that is based on collaborative filtering and on *multi-criteria item ratings*. In such approaches (Adomavicius and Kwon, 2015), users are not expected to specify their preferences for different item features in general but are assumed to rate features of specific items. For example, in the tourism recommendation domain, they might assess a given hotel in dimensions such as value for money, cleanliness, or friendliness of the staff. This more fine-grained preference information can then be used in specifically-extended collaborative filtering approaches, e.g., Adomavicius and Kwon (2007) and Jannach et al. (2012).

A different way to take into account the often multi-faceted nature of individual user preferences is called *calibration*. In these approaches, the idea is not to find items that match user preferences in certain item-specific dimensions but to match past user preferences with respect to certain meta-level properties of the recommendation lists such as diversity. For instance, if for a user of a video streaming platform, interest in various genres was observed, a calibrated recommender system may try to generate a set of item suggestions that reflects this diversity of the user interests.

In an early work, Oh et al. (2011) tried to align the recommendations with the past popularity tendencies of a user where the authors tried to rerank the recommendation lists such that the distribution of the popularity of items in the recommended list to each user, matches their historical tendency toward such items. Later, Jugovac et al. (2017) extended the approach for multiple optimization objectives where authors tried to jointly optimize the relevance of the recommended items along with some additional quality factors such as list diversity, item popularity, and item release years. A more formal characterization of the calibration was introduced by Steck in Steck (2018) who proposed an approach for reranking the recommendations such that the final list is both relevant and also matched the genre preference of the users. Similarly, Abdollahpouri et al. (2021) represents another recent work in that direction where authors aim to tackle the popularity bias problem in recommender systems by reranking the recommendation lists generated for each user such that it has both high relevance and is also in line with the historical popularity tendency of the users.^4^ Overall, in most cases, the central idea of calibration approaches is to match two distributions of some aspect of the recommended items. An alternative optimization goal was used in Jannach et al. (2015a) for the music domain, where the objective was to find musically coherent playlist continuations while preserving prediction accuracy.

#### 2.2.2. Aggregate level

The majority of published research on balancing different recommendation quality aspects targets the aggregate level. The objective of such works is to balance the recommendations for the entire user base, the corresponding metrics are therefore usually averages.^5^ The most common beyond-accuracy measures in the literature include diversity, novelty, serendipity, catalog coverage, popularity bias, or fairness, see, e.g., Adomavicius and Kwon (2012), Kaminskas and Bridge (2016), Vargas and Castells (2011), Abdollahpouri et al. (2017), and Ekstrand et al. (2022). Most commonly, the goal is to balance accuracy with exactly one of these measures, assuming that there is a trade-off between these quality factors. Increasing diversity is for example commonly assumed to have a negative impact on accuracy metrics. A few works exist which consider more than two factors. In an earlier work in this area (Rodriguez et al., 2012), the authors describe an effort to build a *talent recommendation* system at LinkedIn, which not only considers the semantic match between a candidate profile and a job but which also takes side constraints into account, for instance, the presumed willingness of a candidate to change positions. The authors leveraged a constraint-based optimization technique to solve that problem.

Technically, a variety of approaches to balance competing goals can be found in the literature. Reranking accuracy-optimized lists is probably the most common technique and was also used in early approaches for diversification in recommender systems (Bradley and Smyth, 2001). In this work, the particular goal was to diversify the recommendations returned by a *content-based* (case-based) system, which by design are similar to mostly non-diverse results. Notably, to quantify the diversity of a given list, the authors relied on a metric which was later on called *intra-list diversity* in the literature. Technically, three different diversification strategies (randomized, optimizing, greedy) were proposed and evaluated in their work. Generally, reranking techniques were applied in earlier information retrieval settings, in particular in the form of Maximal Marginal Relevance re-ranking (Carbonell and Goldstein, 1998). Since optimal re-ranking strategies are often computationally complex, heuristic or greedy approaches are more common in the literature, e.g., Adomavicius and Kwon (2012), Jugovac et al. (2017), and Abdollahpouri et al. (2021).

An alternative technical approach is taken in Jambor and Wang (2010), where the authors propose a framework based on *constrained linear optimization* to balance potentially competing optimization goals. Their framework primarily considers the assumed *utility* of an item for a given user (e.g., based on a predicted rating), but can also take additional constraints into account in the optimization process. Two example use cases are discussed, (a) promoting long-tail items and (b) the consideration of resources constraints, e.g., stock availability. Experimental evaluations indicate that balancing the trade-offs can be achieved with limited loss in accuracy. As in many other works, the main question however remains how to determine the right trade-off threshold in practice.

An optimization-based method was also proposed in Zhang and Hurley (2008), here with the objective of diversifying the recommendations through a side constraint while maintaining accuracy. The authors propose three ways of formulating the problem. One first possible objective of the optimization task was formulated as to maximize the diversity of the recommendation set while ensuring that the “matching value” (i.e., the preference match or utility for the user) does not fall beyond some tolerance value. An alternative formulation could be to maximize utility while reaching a certain level of diversity. Finally, a problem formulation with a combined optimization goal with a weighting parameter is possible as well. This last suggested problem formulation can be modeled as a binary quadratic programming problem with linear constraints, and the authors present a corresponding solution in their paper.

The *Auralist* framework proposed in Zhang et al. (2012) is designed to deliver not only relevant but also diversified and serendipitous music recommendations. Differently from optimization-based approaches, it works by combining the output of different ranking strategies: an accuracy-based one, one which promotes artists with diverse leadership, and one designed to help users break out of their personal music bubbles. A related approach of combining algorithms with different characteristics is proposed also in Ribeiro et al. (2015). In this latter work, an evolutionary algorithm is used to find a Pareto-efficient hybrid of the different algorithms. While the work in Ribeiro et al. (2015) is only assessed through offline experiments, the authors of *Auralist* evaluated their framework both offline and with the help of a user study. One key insight of the experiments is that serendipitous recommendations indeed lead to higher user satisfaction, despite a certain trade-off in accuracy that was observed in the offline experiments.

A comparison of offline results and a user-centric evaluation is also reported in Said et al. (2013). Here, the authors modified the traditional user-based nearest-neighbor method to consider the ratings of the most distant (“furthest”) neighbors for the predictions. Offline experiments showed that this modification may lead to a notable performance drop in offline experiments. The user study however revealed that the modification did not negatively impact the perceived usefulness of the recommendations, even though they were very different in various dimensions (e.g., novelty, obviousness) than those provided by the traditional algorithm.

More sophisticated, graph-based algorithms for balancing accuracy and other factors, including diversity, were proposed in Zhou et al. (2010) and Isufi et al. (2021). In Zhou et al. (2010), a “heat-spreading” algorithm is applied to the graph formed based on the user-item interaction data. Like in several other works, the authors examine the trade-off between accuracy and other aspects through offline experiments^6^. Isufi et al. (2021) propose a graph convolution approach, building on ideas from Said et al. (2013) discussed earlier, and which only relies on rating information in the recommendation process. Again, offline experiments are conducted to study the accuracy-diversity (and coverage) trade-off.

An alternative technical approach to balance accuracy and novelty is put forward in de Souza Pereira Moreira et al. (2019). In this work, the authors present a generic meta-architecture for news recommendation problems, an application setting where the novelty of the items is often highly related to their relevance. Technically, the use of a parameterizable two-element loss function is proposed, where one part of the loss function targets accuracy and the other novelty. A streaming-based offline evaluation protocol is used to simulate real-world scenarios, and the effects of different hyperparameter settings for the loss function on the accuracy-novelty trade-off are studied.

Finally, McInerney et al. (2018) study the well-known explore-exploit dilemma in recommendation, where the system has the option to either recommend items of which it is relatively sure the user will like, or to take a more risky action and recommend items that should help the system to learn more about the user's preferences. In the latter case, exploring can be seen as taking a chance on an item with the hope that the user will actually like it. One possible problem when only exploiting is that the recommendations can be of limited novelty, and ultimately lead to limited user satisfaction in the long run. In their work, the authors study a contextual bandit approach in the music domain, which also involved the presentation of explanations to the users. Offline experiments on real-world logged interaction data and a partially restricted A/B test provide solid indications for the practical usefulness of the approach.

#### 2.2.3. Discussion

In many cases, optimizations performed at one level, such as the individual level, may affect the other level and vice versa. For example, when calibrating the recommendations for a user to match their *individual* diversity preferences, this will also be reflected to a certain extent on common diversity measures like intra-list diversity, when measured at the population-wide (*aggregate*) level. However, the relationship between individual-level and aggregate-level optimizations and the resulting effects may however not always be trivial in nature. Klimashevskaia et al. (2022), for example, found that calibrating recommendations with respect to popularity had a clear impact on the recommendations lists for *some users*, but it was found that the desired aggregate effect of reducing the popularity bias of the recommendations across users was not as substantial as expected. Similar considerations can be made for other quality objectives. For example, when optimizing recommendations for the individual user's *value-for-money* objective, this may have an impact on the overall revenue at the aggregate level. In sum, it therefore often seems advisable to observe multiple metrics in parallel to be able to understand the potentially subtle relationships between individual optimization goals.

### 2.3. Multistakeholder objectives

The beyond-accuracy quality metrics discussed in the previous section were historically mostly introduced to improve recommendations for end users. Higher diversity, for example, should avoid monotonicity, and novelty should support discovery. The underlying assumption—also of pure accuracy-oriented works—is that improving different quality aspects for users would be the sole factor for a successful recommender. Only in recent years, more attention has been paid in the literature to the fact that many recommendation scenarios in the real world are situated in environments, where the objectives of multiple stakeholders have to be considered. The common players in such *multistakeholder* recommendation problems include *end consumers*, the recommendation *platform*^7^, item *providers* (suppliers), and sometimes even parts of a broader *society* (Abdollahpouri et al., 2020; Jannach and Bauer, 2020). In such settings, a recommender system may serve different purposes for different stakeholders (Jannach and Adomavicius, 2016), and the related objectives may stand in conflict.

In some cases there may even be subgroups within the consumer stakeholder group that have to be considered. These subgroups may have different expectations when using the service, and a recommender system should take these into account. In the music domain, for example, there can be different types of consumers, where one group's goal might lie in the exploration of the catalog and another group might be more interested in mood enhancement, see Bogt et al. (2011). The corresponding algorithms should then try to take the users' goals appropriately into account, see also Kapoor et al. (2015). Subgroups in a consumer stakeholder group can however also be identified by the providers, e.g., free vs. premium or new vs. existing customers, for which different objectives may exist. A number of recent research works in particular in the area of *fair recommender systems* address this latter problem. In Li et al. (2021) and Wu et al. (2022), for example, the authors investigate if highly active and less active users (including cold-start users) receive recommendations of largely different quality.

A typical problem setting in practice that involves *two stakeholders* is that of balancing consumer and platform objectives. In many cases, there may be a potential trade-off between (a) recommending the *most relevant* items for consumers and (b) recommending items that are also somewhat relevant but assumed to be favorable in terms of the platform's business objectives^8^. Some of the discussed beyond-accuracy metrics can actually be seen as serving both stakeholders. Making more novel recommendations not only potentially leads to a better user experience, but also to more engagement with the service and longer-term customer retention, which is an important platform goal in many application contexts (Anderson et al., 2020).

A number of research works however also consider monetary more directly, in particular in the form of recommender systems that are “price and profit aware.” For example, Jannach and Adomavicius (2017) proposes a simple profit-aware recommendation approach *via* a simulation on a movie dataset by incorporating purchase-oriented information such as the price of the movie, sales probabilities, and the resulting profit, and shows that the approach can generate recommendations with yield higher profit with minimum loss in the relevance of the recommended movies. In Chen et al. (2008), as another work, two heuristic profit-aware strategies are proposed and the authors found that such methods can increase the profit from cross-selling without losing much recommendation accuracy.

Following a quite different technical approach, Wang and Wu (2009) develop an analytical model and optimization-based framework, which allows to *numerically study* the (short-term) effects of different marketing strategies. Possible strategies for example include a profit maximization approach or a “win-win” strategy for the platform and for consumers. The underlying model not only considers the relevance of the items that can be recommended to users, but also the items' selling price and profit. Moreover, budget constraints on the consumers' side are modeled as well. To address the challenges of fast online recommendation, an efficient solving strategy is proposed.

Differently from the works discussed so far, Azaria et al. (2013) investigate the effects of profit-aware and “value-aware” recommendation strategies through a *user study*. Two strategies are proposed which can be applied on top of any black-box recommendation model. In one strategy (“Hidden Agenda”), no prices for the items are present, whereas in the other (“Revenue Maximizing”) sales prices are considered. In the user study, participants received personalized recommendations and were then informed, among other aspects, about their satisfaction with the recommendation and their willingness to pay (WTP) for individual movies. The results show that the developed strategies can markedly increase the profit of the platform without a measurable drop in user satisfaction.

The results from a *field study* in the form of a *randomized controlled trial* are reported in Panniello et al. (2016). The specific goal of the study was to investigate the consumers' reactions in terms of purchasing behavior and (long-term) trust when confronted with recommendations that aim to balance accuracy and profitability. The experimental design included a profit-aware algorithm and a profit-agnostic one, and the recommendations were delivered to customers through personalized newsletters. The analyses after a 9-week period showed that higher profit can be achieved without a loss in consumer trust. Moreover, it turned out that the profit gains could be attributed to a combination of factors, consumer trust, diversity, and the relevance of the recommendations.

Besides situations with potential trade-offs at the recommendation platform side, there is the specific setting of *group recommendation*, a problem that has been studied for several years, even though not under the name multistakeholder recommendation (Masthoff, 2015). In such settings, the system's goal is to determine a set of recommendations that suit the preferences of a group of users, e.g., friends who want to watch a movie together. A unique aspect of such settings is that all involved (consumer) stakeholders in some ways receive or have to accept the same recommendation, which may or may not fit their preferences very well. A variety of strategies to aggregate individual user preferences were proposed over the years. Early works on the topic can be found in O'Connor et al. (2001) and Masthoff (2004). In Masthoff (2004), for instance, Masthoff reports the outcomes of different user studies aimed to understand how humans make choices for a group and find that humans indeed sometimes follow strategies inspired by Social Choice Theory (Sen, 1986). We iterate here that the group recommendation setting differs from other multistakeholder scenarios in that all stakeholders receive the same set of recommendations.

*Reciprocal recommendation* is another specific set of problem settings involving multiple stakeholders. Here, instead of recommending items to users, the problem is to recommend users to users, also known as people-to-people recommendation. Typical application scenarios are recommendations on dating (Pizzato et al., 2010) and recruiting platforms (Siting et al., 2012). A particularity of such settings is that the success of a recommendation is not determined solely by the recipient of the recommendation, but there must be a mutual preference match or compatibility between the two people involved, see Palomares et al. (2021) for an in-depth discussion on the topic. The recommendation platform (service provider), therefore, faces additional complexities in the matching process and in parallel has to observe its own business objectives and constraints. On a job recommendation platform, for example, the platform may have to additionally ensure that each paid job advertisement receives a minimum number of relevant impressions, i.e., exposure (Abel et al., 2017).

Similar considerations may generally apply when the recommendation platform serves as a marketplace with multiple suppliers of identical or comparable items. Let us consider again the example of a typical hotel booking platform, which serves personalized recommendations to its users (Jannach and Bauer, 2020). Besides the consumer, who already might have competing objectives, there are the property owners, who have their offerings listed on the booking platform and pay a commission for each booking. The goal of the property owners is that their offerings are exposed to as many matching customers as possible in order to increase the chances of being booked. The booking platform, finally, may not only be interested in recommending matching hotels to consumers but might also seek to maximize their commission, e.g., by recommending slightly more expensive hotels. In addition, to balance these objectives, the platform may furthermore have to ensure that *all* listed properties reach a sufficient level of exposure, i.e., chance of being booked. This may be required to ensure a long-term relationship with property owners, who might otherwise discontinue listing their offerings on the platform at some stage (Krasnodebski and Dines, 2016).

### 2.4. Time horizon objectives

In some application domains, it might be quite simple to increase short-term Key Performance Indicators. In the hotel booking scenario which we have just discussed, boosting short-term revenue might be achieved by recommending hotels with currently discounted rates, which maximizes the probability of a transaction (Jannach et al., 2017). In the news domain, recommending articles on trending topics, articles with click-bait headlines, or generally popular content such as celebrity gossip may lead to high click-through rates (CTR). In the music domain, recommending tracks of trending or popular artists, which the user already knows, might be a safe strategy when the target metric is to avoid “skip” events.

Such strategies that are successful in the short term may however be non-optimal or even detrimental in the long run. The recommendation of discounted hotel rooms may be bad for profit, and recommending hotels that lead to the highest commission may hurt consumer trust. News readers may be disappointed when actually reading articles with a click-bait headline and may not trust these recommendations in the future. Music listeners finally may have difficulties discovering new artists over time and may quit using the service after some time.

Most academic research is based on one-shot evaluations, typically focusing on prediction accuracy given a static dataset and a certain point in time. The longitudinal effects of different recommendation strategies are much less explored and there is also limited literature on the long-term effects of recommender systems in the industry. A/B tests in the industry may last from a few weeks to several months. In Gomez-Uribe and Hunt (2015), the case of Netflix is discussed, where one main KPI is customer retention, which is oriented toward the long-term perspective. In their case, attributing changes in the recommender system to such long-term effects is reported to be challenging, e.g., because of already high retention rates and the need for large user samples. Other reports from real-world deployments of recommender systems can be found in Panniello et al. (2016) or Lee and Hosanagar (2019). In Lee and Hosanagar (2019), the authors for example found that using a recommender system led to decreased sales diversity compared to a situation without a recommender.^9^ A similar effect was reported in Anderson et al. (2020), where the recommender system on a music streaming site led to a reduced aggregate consumption diversity. A survey of other reports on real-world applications of recommender systems can be found in Jannach and Jugovac (2019).

Given the limitations of one-shot evaluations, we have observed an increased interest in longitudinal studies in recent years. One prominent line of research lies in the area of reinforcement learning (RL) approaches in particular in the form of contextual bandits, see e.g., Li et al. (2010) for earlier work in the news domain. In such approaches, the system sequentially selects items to recommend to users and then incorporates the users' feedback for subsequent recommendations. Different recommendation algorithms can be evaluated offline with the help of simulators, e.g., Rohde et al. (2018) and McInerney et al. (2021). A common challenge in this context is to ensure that such evaluations are unbiased (Li et al., 2011; Huang et al., 2020).^10^ We note that the consideration of temporal aspects such as different time horizons or delayed feedback has been explored in the RL literature for the related problem of computational advertising for several years (Chapelle, 2014; Theocharous et al., 2015).

Reinforcement learning approaches typically aim at finding a strategy to maximize the expected *reward*. During the last few years, a number of studies that use *other forms* of simulations were published that focus on other important long-term phenomena of recommender systems. These studies for example focus on longitudinal effects of recommender systems on sales diversity (Fleder and Hosanagar, 2009), potential reinforcement effects in terms of popularity bias, and other aspects for traditional and session-based recommendations (Jannach et al., 2015b; Ferraro et al., 2020), longitudinal performance effects of recommender systems and the “performance paradox” (Zhang et al., 2019), differences in terms of long-term effects of consumer-oriented and profit-oriented recommendation strategies (Ghanem et al., 2022).

Directly optimizing for long-term rewards is typically hard due to the sparsity in observing these events and the low signal-to-noise ratio (weak connection) between these long-term outcomes and a single recommendation. Therefore, researchers often leverage surrogates or mid-level outcomes that are easier to observe as a proxy for potential long-term outcomes. For example, Wang et al. (2022) investigates several surrogates such as diversity of consumption, frequency of returning to the platform, repeated consumption, etc., as a proxy to estimate long-term user engagement. The authors then use such surrogates in the objective function for the RL algorithm to optimize for those metrics. With their work, they aim at providing guidance for researchers and practitioners when selecting surrogate measures to address the difficult problem of optimizing for long-term objectives.

### 2.5. User experience objectives

Going beyond the specifics of individual algorithms, there can be also various objectives to be pursued at the user interaction level of a recommender system. The design space for the user interface of recommender systems is actually large, see Jugovac and Jannach (2017), and there thus may be a number of competing objectives at the user interface (UI) level.

Here, we only list a few examples of potential trade-offs that may be common for many recommender system applications.

- *Information completeness vs. information overload*: This, for instance, refers to the question of how many items should be shown to users and if we should completely filter out certain items from the result list. Showing too few options may give users the feeling that the system holds back some information. If there is too much information users will find themselves again in a situation of information overload (Bollen et al., 2010; Aljukhadar et al., 2012). Besides the question of how many options to show, a related question is how much detail and additional information to show for each recommendation.
- *Transparency and user control vs. cognitive effort*: Transparency and explanations are commonly considered to be trust-establishing factors in recommender systems (Pu et al., 2011). A variety of different ways of explaining recommendations were proposed in the literature (Tintarev and Masthoff, 2011; Nunes and Jannach, 2017). Many of these academic proposals are quite complex and may easily cognitively overload average end users. Similar considerations apply for approaches that implement mechanisms for *user control* in recommender systems (Ekstrand et al., 2015; Jannach et al., 2016).
- *Flexibility vs. efficiency*: This question arises in the context of modern conversational recommender systems that are implemented in the form of chatbots. Chatbots typically support two forms of interactions: a) natural language input and b) form-based input (i.e., using buttons). While natural language inputs may allow for more flexible interactions, the study in Iovine et al. (2020), for instance, indicated that a combination of interaction modalities was most effective.

Several other more general design trade-offs may exist depending on the specific application, e.g., regarding acceptable levels of automating adaptivity of the user interface, which may hamper usability (Paymans et al., 2004).

### 2.6. Engineering objectives

In this final category, we discuss technical aspects and their potential trade-offs. We call them “engineering objectives”, as they refer to more general system properties.

One such trade-off in practice may lie in the complexity of the underlying algorithms and the gains that one may obtain in terms of business-related KPIs. Already in the context of the Netflix Prize (Bennett and Lanning, 2007) we could observe that the winning solutions were finally not put into production, partly due to their complexity. Similar considerations can be made for today's sometimes computationally demanding methods based on deep learning. In some cases, there might be a diminishing return on deploying the most sophisticated models in production, only because they lead to slightly better accuracy values in offline testing. In some research works, it even turns out that “embarrassingly shallow” models can be highly competitive in offline evaluations (Steck, 2019).

With highly complex models, not only scalability issues may arise and monetary costs for computing resources may increase, but the complexity of the architectures might also make such systems more difficult to maintain, debug, and explain. On the other hand, solutions built upon modern deep learning frameworks are sometimes reported to be advantageous over conceptually simpler, but specialized solutions, because these frameworks and deep learning architectures make it very easy to integrate various types of information into the models (Steck et al., 2021).

However, integrating different types of information can also come at a price. In many organizations, the different pieces of information that should be integrated into a recommender system—e.g., user behavior logs, purchase records, item meta-data, stock availability, and business rules—may be stored in various systems and databases. This can make data integration and data quality assurance a highly challenging task, in cases where increasingly more data sources must be combined.

## 3. Summary and challenges

Our review outlines that providing automated recommendations is a problem that may require the consideration of more than one objective in many real-world use cases. Such multi-objective settings may include competing objectives of consumers, possible tensions between the goals of different stakeholders, conflicts when optimizing for different time horizons, competing design choices at the UI level, as well as system-level and engineering-related considerations. In this work, we reviewed the literature in this area and provided a taxonomy to organize the various dimensions of multi-objective recommendation. We note here that the categories of the taxonomy are not mutually exclusive. For instance, a multi-objective recommendation approach may address both aspects regarding different time horizons as well as the possibly competing goals of the involved stakeholders.

In practice, one main challenge may usually lie in deciding on the right balance between the competing goals from an organizational perspective. Various stakeholders from different organizational units may have to agree on such decisions, and corresponding KPIs need to be defined and monitored. Given these KPIs, suitable optimization goals and possibly proxy measures have to be implemented and validated at the technical level.

In academic settings, researchers typically abstract from the specifics of a given application context, aimed at developing generalizable algorithmic solutions to deal with multi-objective problem settings. This abstraction process commonly involves the use of *offline evaluation approaches*, the establishment of certain assumptions, and the introduction of computational metrics which should be optimized. After such an abstraction, one main challenge, however, lies in the evaluation process and, in particular, in making sure that improvements that are observed in terms of abstract evaluation measures would translate to better systems in practice (Cremonesi and Jannach, 2021).

Unfortunately, in many of today's research works, we observe phenomena similar to the “abstraction traps” described by Selbst et al. (2019) in the context of research on algorithmic works in *Fair Machine Learning*. In the case of competing individual-level quality goals, for example, how can we be sure that a particular diversity metric, which we optimize such as an intra-list similarity, matches human perceptions and what would be the right balance for a given application setting or an individual user? How do we know if calibrated recommendations are liked more by users, and what would be the effects of calibration on organizational goals? Answering such questions requires corresponding user studies to, e.g., validate that the computational metrics are good proxies for human perceptions. An attempt to investigate the relationship between *perceived diversity* and the widely used intra-list similarity measure can be found in Jesse et al. (2022).

The problem however becomes even more challenging when not even the target concepts are entirely clear. In recent years, a widely investigated multi-objective problem setting is the provision of *fair* recommendations (Ekstrand et al., 2022). Unfortunately, optimizing for fairness turns out to be challenging, as fairness is a societal construct, and a number of definitions exist, see Narayanan (2018). Researchers in computer science, therefore, came up with various types of ways of operationalizing fairness constraints. However, in many of such works, little or no evidence or argumentation is provided why the chosen fairness metrics are meaningful in practice in general or in a particular application setting, see Deldjoo et al. (2022) for a survey on the recent literature.

In some cases, including our own previous work, e.g., Abdollahpouri et al. (2019a), making fair recommendations is only loosely connected or even simply equated with reducing the popularity bias of recommendations. Technically, this is often done by matching it with a target distribution or metric threshold, which is assumed to be given. In reality, however, it is not clear what would be the underlying *normative claim* that mandates that less popular items should be recommended. In fact, many of these unpopular items might simply be of poor quality. Moreover, users might not even *perceive* such recommendations of unpopular items to be fair. However, there are also studies that indicate that recommending mostly popular items may negatively impact accuracy, and, importantly, that these effects may differ across user groups. Our previous study in the movie domain (Abdollahpouri et al., 2019b), for example, indicated that users of the group with the least mainstream taste received the worst recommendations. A similar observation was later made in the music domain by Kowald et al. (2020). We note that here, item popularity is often assessed by counting the number of past interactions in the database. The assumed fairness problem is thus related, but different from the *item cold-start* problem (Panda and Ray, 2022). Recommending such items is of course important in practice, to ensure a certain level of initial exposure to new items.

Overall, these observations call for more studies involving humans in the evaluation loop and industry partners in the research process. However, only a few works exist in that direction so far. An example of a user study can be found in Azaria et al. (2013), and outcomes of field studies are described in Panniello et al. (2016). An offline evaluation with real-world data from the industry is done in Mehrotra et al. (2018), but even in this case, it is not clear if the computational metrics truly correspond to the real-world goals, e.g., if more listening events on the music platform lead to higher user satisfaction as claimed.

Ultimately, despite such recent progress, multi-objective recommender systems remains a highly important research area with a number of challenging research questions. Addressing such questions will however help to pave the way toward more impactful recommender systems research in the future.

## Author contributions

DJ and HA: conceptualization, research, and writing. All authors contributed to the article and approved the submitted version.

## Footnotes

^2^A prime example in this context is that Netflix never put the winning solution of their Netflix Prize Challenge into production, see https://www.wired.com/2012/04/netflix-prize-costs/.

^3^See also He et al. (2016) and Jugovac and Jannach (2017) for surveys on interactive recommender systems.

^4^While such a calibration approach turns out to be effective to mitigate popularity bias on the individual level, it may be limited in terms of reducing this bias across an entire user population (Klimashevskaia et al., 2022).

^5^We acknowledge that the distinction between individual level and aggregate level can be viewed from different perspectives for calibration approaches where often also overall effects of user-individual calibration effects are reported, e.g., in the average reduction of the gap between the user profile and recommendation characteristics (Jugovac et al., 2017).

^6^While the authors claim to “solve” the accuracy-diversity dilemma, they technically propose specific measures to gauge the level of personalization and novelty of the recommendations. Their definition of diversity is not depending on item features and is thus rather uncommon in the literature.

^7^This is sometimes called the *service providers* in the literature.

^8^See Shih and Kaufmann (2011) for a discussion of Netflix DVD recommendation strategy in 2011, which aimed to promote items that are less costly than blockbusters.

^9^It is worth noting that the authors studied one particular class of non-personalized recommendation algorithms here based on co-purchasing statistics (“Customers who bought this item also bought …”).

^10^A critical discussion of current evaluation practices when applying RL for sequential problems can be found in Deffayet et al. (2022).
